# Supplementary material for: Most science is published from countries lacking in democracy and freedom of press
Source: Res Integr Peer Rev. 2026 Feb 5;11:4. doi: 10.1186/s41073-026-00190-6 (PMC12874684; doi:10.1186/s41073-026-00190-6)

**SUPPLEMENT: Supplementary Data and Supplementary Figure 1**

**Supplementary Data**

Number of publications in Scopus in 2006 and 2024 (fractional count)

| **Country** | **2006** | **2024** | **2006 Established** | **2024 Established** | **2024 New continuous** | **2006 Elliptical** | **2024 Elliptical** |
| --- | --- | --- | --- | --- | --- | --- | --- |
| China | 186,060.3 | 1,129,320.2 | 132,447.5 | 609,824.7 | 387,612.4 | 53,612.8 | 131,883.1 |
| United States | 459,524.2 | 548,583.9 | 309,732.5 | 282,289.0 | 150,321.9 | 149,791.7 | 115,973.0 |
| India | 41,162.3 | 311,561.4 | 30,206.1 | 110,446.0 | 103,035.5 | 10,956.2 | 98,079.8 |
| United Kingdom | 111,586.5 | 143,768.1 | 76,654.4 | 71,892.0 | 39,780.6 | 34,932.0 | 32,095.5 |
| Germany | 97,194.1 | 132,663.1 | 65,098.5 | 66,769.5 | 40,459.1 | 32,095.7 | 25,434.6 |
| Italy | 54,058.7 | 109,431.2 | 38,245.3 | 49,833.0 | 41,619.4 | 15,813.5 | 17,978.8 |
| Japan | 117,026.8 | 108,010.8 | 78,623.0 | 57,567.8 | 33,078.5 | 38,403.7 | 17,364.6 |
| Russia | 29,549.3 | 89,783.2 | 23,884.4 | 33,142.6 | 44,023.3 | 5,664.9 | 12,617.3 |
| South Korea | 38,399.2 | 85,238.2 | 28,505.4 | 37,849.9 | 37,596.8 | 9,893.8 | 9,791.5 |
| Spain | 42,342.2 | 82,937.5 | 31,969.9 | 37,462.7 | 35,468.8 | 10,372.3 | 10,006.0 |
| Canada | 58,421.1 | 79,641.5 | 40,351.3 | 42,190.4 | 22,867.7 | 18,069.8 | 14,583.4 |
| France | 66,332.3 | 74,688.9 | 45,518.5 | 41,007.1 | 20,714.8 | 20,813.9 | 12,967.0 |
| Australia | 39,843.0 | 73,488.6 | 27,819.9 | 40,455.6 | 21,043.6 | 12,023.1 | 11,989.4 |
| Brazil | 29,468.8 | 73,303.7 | 22,593.1 | 38,332.8 | 27,370.8 | 6,875.7 | 7,600.1 |
| Turkey | 20,412.1 | 64,299.9 | 15,631.5 | 33,631.9 | 22,607.3 | 4,780.6 | 8,060.7 |
| Iran | 10,688.7 | 63,764.0 | 6,924.2 | 30,168.6 | 28,786.9 | 3,764.5 | 4,808.6 |
| Indonesia | 858.2 | 57,597.3 | 505.9 | 13,169.4 | 35,497.9 | 352.3 | 8,929.9 |
| Poland | 22,911.8 | 44,575.1 | 16,450.2 | 18,434.1 | 20,690.4 | 6,461.5 | 5,450.7 |
| Netherlands | 29,319.6 | 42,483.3 | 21,060.8 | 23,119.9 | 12,191.2 | 8,258.7 | 7,172.3 |
| Saudi Arabia | 2,191.5 | 35,514.7 | 1,455.4 | 14,083.8 | 17,731.9 | 736.1 | 3,699.0 |
| Malaysia | 3,555.5 | 33,685.1 | 1,939.4 | 10,578.0 | 17,390.7 | 1,616.1 | 5,716.4 |
| Taiwan | 26,876.6 | 33,175.7 | 18,533.2 | 17,166.7 | 10,894.7 | 8,343.4 | 5,114.3 |
| Switzerland | 20,063.6 | 30,357.9 | 14,248.2 | 15,662.4 | 9,410.9 | 5,815.4 | 5,284.7 |
| Egypt | 4,354.3 | 28,607.1 | 3,009.2 | 13,233.4 | 12,029.6 | 1,345.2 | 3,344.1 |
| Sweden | 18,528.9 | 27,714.3 | 13,900.5 | 14,671.1 | 8,514.0 | 4,628.3 | 4,529.3 |
| Pakistan | 2,979.6 | 25,437.0 | 2,109.6 | 12,172.9 | 10,148.5 | 870.0 | 3,115.6 |
| Mexico | 10,098.7 | 25,087.1 | 7,075.3 | 10,534.2 | 10,879.1 | 3,023.4 | 3,673.9 |
| Portugal | 7,814.8 | 24,483.0 | 5,388.5 | 9,509.2 | 11,212.0 | 2,426.3 | 3,761.8 |
| South Africa | 7,001.2 | 23,749.2 | 5,068.4 | 9,085.3 | 9,860.2 | 1,932.8 | 4,803.7 |
| Belgium | 15,103.9 | 22,419.2 | 10,546.1 | 12,079.6 | 6,748.2 | 4,557.8 | 3,591.4 |
| Thailand | 4,709.6 | 22,336.3 | 3,218.0 | 7,051.6 | 12,228.4 | 1,491.5 | 3,056.3 |
| Iraq | 279.8 | 21,392.9 | 151.1 | 6,506.0 | 12,304.9 | 128.7 | 2,582.0 |
| Hong Kong | 10,145.3 | 21,083.5 | 7,143.3 | 11,239.5 | 6,071.5 | 3,002.0 | 3,772.6 |
| Ukraine | 5,426.4 | 19,638.1 | 3,675.2 | 4,659.3 | 12,259.1 | 1,751.2 | 2,719.7 |
| Denmark | 9,600.1 | 19,508.4 | 7,237.7 | 10,908.4 | 5,540.7 | 2,362.4 | 3,059.3 |
| Austria | 10,516.6 | 18,024.3 | 7,197.8 | 8,840.7 | 5,807.5 | 3,318.8 | 3,376.1 |
| Norway | 8,079.6 | 17,784.0 | 5,938.5 | 9,379.9 | 5,299.1 | 2,141.1 | 3,105.1 |
| Greece | 11,783.6 | 17,730.5 | 8,434.3 | 6,655.1 | 8,005.9 | 3,349.3 | 3,069.5 |
| Israel | 12,640.4 | 17,679.3 | 9,519.9 | 9,699.2 | 5,189.2 | 3,120.4 | 2,790.9 |
| Czech Republic | 9,487.4 | 17,234.9 | 6,742.3 | 8,352.2 | 6,494.5 | 2,745.1 | 2,388.2 |
| Vietnam | 495.1 | 16,166.4 | 346.0 | 5,492.1 | 8,132.6 | 149.2 | 2,541.7 |
| Finland | 11,450.6 | 16,044.7 | 7,637.6 | 8,261.4 | 4,519.5 | 3,813.0 | 3,263.8 |
| Singapore | 9,289.4 | 15,636.8 | 6,220.8 | 7,296.2 | 4,691.9 | 3,068.6 | 3,648.7 |
| Romania | 4,135.1 | 14,880.2 | 2,413.6 | 4,070.4 | 8,270.7 | 1,721.5 | 2,539.1 |
| Morocco | 1,070.8 | 14,763.3 | 722.4 | 4,441.8 | 7,985.0 | 348.5 | 2,336.5 |
| Nigeria | 2,911.1 | 13,575.8 | 1,365.5 | 3,739.8 | 6,954.5 | 1,545.6 | 2,881.5 |
| Chile | 3,292.4 | 12,639.9 | 2,522.7 | 5,337.8 | 5,894.9 | 769.8 | 1,407.1 |
| Colombia | 1,598.4 | 12,396.5 | 1,160.9 | 4,051.0 | 6,683.5 | 437.5 | 1,662.0 |
| Hungary | 5,922.0 | 11,620.8 | 4,232.8 | 4,505.7 | 5,200.9 | 1,689.2 | 1,914.1 |
| Ireland | 5,791.2 | 11,550.7 | 3,966.2 | 5,763.1 | 3,394.7 | 1,825.0 | 2,393.0 |
| Argentina | 6,003.8 | 11,539.2 | 4,506.8 | 5,711.8 | 4,291.6 | 1,497.0 | 1,535.9 |
| United Arab Emirates | 971.5 | 11,183.5 | 526.4 | 3,482.6 | 4,518.5 | 445.1 | 3,182.5 |
| Bangladesh | 887.1 | 11,084.9 | 491.0 | 2,814.5 | 5,267.2 | 396.1 | 3,003.1 |
| New Zealand | 6,799.7 | 10,392.1 | 5,026.1 | 5,609.3 | 2,789.1 | 1,773.6 | 1,993.7 |
| Algeria | 1,433.9 | 10,209.6 | 731.6 | 3,928.7 | 4,250.4 | 702.3 | 2,030.5 |
| Ethiopia | 311.2 | 9,116.4 | 244.7 | 3,075.9 | 5,509.8 | 66.5 | 530.7 |
| Jordan | 1,061.8 | 8,369.2 | 742.3 | 2,457.2 | 4,802.5 | 319.5 | 1,109.5 |
| Peru | 576.1 | 8,339.4 | 476.2 | 1,650.8 | 5,517.7 | 99.8 | 1,170.9 |
| Tunisia | 2,078.0 | 7,764.0 | 1,363.4 | 3,044.7 | 3,520.9 | 714.6 | 1,198.4 |
| Serbia | 2,358.1 | 7,686.2 | 1,582.1 | 2,842.5 | 3,604.0 | 776.0 | 1,239.7 |
| Philippines | 532.3 | 7,270.8 | 379.1 | 1,541.0 | 3,213.4 | 153.2 | 2,516.3 |
| Croatia | 3,245.5 | 6,989.4 | 2,180.2 | 2,291.1 | 3,457.9 | 1,065.3 | 1,240.5 |
| Kazakhstan | 242.3 | 6,811.2 | 190.6 | 1,510.7 | 4,427.8 | 51.7 | 872.7 |
| Slovakia | 2,787.0 | 6,754.9 | 2,141.5 | 2,354.7 | 3,302.0 | 645.4 | 1,098.1 |
| Bulgaria | 2,021.6 | 6,471.3 | 1,336.2 | 1,738.2 | 2,939.0 | 685.4 | 1,794.1 |
| Uzbekistan | 334.1 | 5,779.2 | 275.5 | 1,773.1 | 3,377.4 | 58.6 | 628.7 |
| Ecuador | 151.9 | 5,661.4 | 105.9 | 1,231.9 | 3,703.6 | 45.9 | 725.8 |
| Slovenia | 2,709.1 | 5,253.4 | 1,996.9 | 2,102.1 | 2,232.2 | 712.2 | 919.1 |
| Ghana | 238.8 | 5,059.7 | 167.8 | 1,700.4 | 2,673.0 | 71.0 | 686.2 |
| Lithuania | 1,968.9 | 3,918.5 | 1,458.2 | 1,247.5 | 2,195.2 | 510.8 | 475.8 |
| Macao | 206.9 | 3,852.5 | 118.0 | 1,945.8 | 1,301.0 | 88.9 | 605.7 |
| Qatar | 214.2 | 3,440.0 | 112.8 | 1,241.1 | 1,475.8 | 101.4 | 723.0 |
| Lebanon | 761.3 | 3,248.5 | 537.7 | 1,430.8 | 1,429.7 | 223.6 | 388.0 |
| Sri Lanka | 444.0 | 3,073.2 | 249.4 | 851.2 | 1,166.8 | 194.6 | 1,055.2 |
| Oman | 405.6 | 2,962.8 | 296.7 | 808.2 | 1,385.3 | 108.9 | 769.4 |
| Cyprus | 553.3 | 2,793.6 | 334.6 | 1,210.9 | 1,035.6 | 218.7 | 547.0 |
| Kenya | 606.1 | 2,705.0 | 459.6 | 883.8 | 1,375.0 | 146.6 | 446.2 |
| Azerbaijan | 314.1 | 2,608.0 | 225.4 | 728.3 | 1,538.0 | 88.7 | 341.7 |
| Estonia | 1,028.7 | 2,482.6 | 694.4 | 950.8 | 974.5 | 334.3 | 557.3 |
| Nepal | 389.4 | 2,314.5 | 273.1 | 880.5 | 1,153.7 | 116.3 | 280.3 |
| Tanzania | 326.9 | 2,229.2 | 239.3 | 766.6 | 1,192.8 | 87.6 | 269.9 |
| Kuwait | 760.0 | 2,209.7 | 504.5 | 755.9 | 1,044.9 | 255.5 | 409.0 |
| Cameroon | 361.4 | 2,180.7 | 268.1 | 940.1 | 1,060.7 | 93.3 | 179.9 |
| Latvia | 431.2 | 2,112.7 | 258.9 | 485.5 | 1,200.5 | 172.3 | 426.7 |
| Uganda | 230.5 | 1,959.0 | 189.7 | 782.3 | 893.3 | 40.7 | 283.4 |
| Luxembourg | 331.3 | 1,753.7 | 178.7 | 724.2 | 515.0 | 152.7 | 514.4 |
| Belarus | 1,257.3 | 1,563.0 | 999.4 | 497.4 | 839.5 | 257.9 | 226.1 |
| Cuba | 1,445.8 | 1,507.7 | 948.4 | 611.7 | 760.7 | 497.4 | 135.3 |
| Uruguay | 394.2 | 1,417.8 | 303.4 | 658.7 | 504.9 | 90.8 | 254.3 |
| Palestinian Territory | 142.5 | 1,417.8 | 105.7 | 451.9 | 798.1 | 36.8 | 167.9 |
| Bosnia and Herzegovina | 320.1 | 1,402.0 | 195.0 | 414.3 | 732.3 | 125.1 | 255.5 |
| Armenia | 396.6 | 1,386.1 | 302.1 | 555.8 | 600.8 | 94.5 | 229.5 |
| Bahrain | 194.6 | 1,269.7 | 139.8 | 259.3 | 546.9 | 54.8 | 463.4 |
| Georgia | 507.7 | 1,186.2 | 398.7 | 395.8 | 531.3 | 109.0 | 259.2 |
| Iceland | 444.6 | 1,071.3 | 343.6 | 520.5 | 344.4 | 101.0 | 206.5 |
| Costa Rica | 263.8 | 1,032.7 | 209.9 | 322.4 | 529.6 | 53.9 | 180.6 |
| Zimbabwe | 196.9 | 995.5 | 136.3 | 235.2 | 407.3 | 60.7 | 353.0 |
| Syria | 116.7 | 953.1 | 79.8 | 258.0 | 588.2 | 36.9 | 106.9 |
| Albania | 34.5 | 862.4 | 22.2 | 124.3 | 590.4 | 12.4 | 147.7 |
| Sudan | 122.7 | 772.8 | 84.7 | 248.9 | 383.4 | 38.0 | 140.6 |
| Malta | 124.2 | 772.1 | 59.5 | 237.0 | 312.8 | 64.6 | 222.2 |
| Kyrgyzstan | 55.4 | 750.8 | 38.3 | 120.5 | 544.5 | 17.1 | 85.8 |
| North Macedonia | 246.7 | 743.4 | 127.6 | 168.8 | 396.2 | 119.0 | 178.4 |
| Yemen | 59.1 | 699.3 | 45.7 | 222.8 | 357.8 | 13.4 | 118.6 |
| Senegal | 163.3 | 682.1 | 77.9 | 224.6 | 360.5 | 85.4 | 96.9 |
| Brunei | 47.9 | 658.9 | 32.5 | 167.2 | 295.7 | 15.4 | 196.0 |
| Venezuela | 1,511.5 | 651.2 | 890.9 | 195.4 | 330.3 | 620.6 | 125.5 |
| Burkina Faso | 112.7 | 649.4 | 80.1 | 229.1 | 342.7 | 32.6 | 77.6 |
| Jamaica | 390.4 | 633.9 | 210.9 | 217.2 | 184.3 | 179.5 | 232.3 |
| Puerto Rico | 691.4 | 631.0 | 486.3 | 269.7 | 264.3 | 205.1 | 96.9 |
| Rwanda | 23.1 | 619.7 | 15.9 | 213.6 | 336.5 | 7.2 | 69.5 |
| Libya | 110.3 | 592.8 | 56.4 | 135.2 | 249.6 | 53.9 | 208.0 |
| Malawi | 110.3 | 581.2 | 86.8 | 200.4 | 258.7 | 23.5 | 122.1 |
| Benin | 76.4 | 573.6 | 44.6 | 198.5 | 316.5 | 31.8 | 58.7 |
| Botswana | 239.5 | 567.9 | 162.9 | 201.4 | 241.2 | 76.6 | 125.3 |
| Zambia | 82.3 | 528.5 | 67.5 | 178.6 | 264.0 | 14.8 | 85.8 |
| Moldova | 173.5 | 480.4 | 136.1 | 164.7 | 238.6 | 37.4 | 77.1 |
| Ivory Coast | 137.9 | 466.1 | 73.4 | 158.3 | 261.2 | 64.5 | 46.5 |
| Democratic Republic of the Congo | 36.7 | 439.3 | 19.7 | 181.5 | 225.3 | 17.0 | 32.5 |
| Mongolia | 60.0 | 395.9 | 38.6 | 151.1 | 184.6 | 21.4 | 60.2 |
| Panama | 37.2 | 375.3 | 28.1 | 83.2 | 164.4 | 9.1 | 127.7 |
| Namibia | 46.7 | 374.8 | 38.3 | 114.5 | 133.2 | 8.4 | 127.1 |
| Paraguay | 39.3 | 363.3 | 20.1 | 99.9 | 210.2 | 19.2 | 53.2 |
| Somalia | 2.0 | 352.5 | 2.0 | 77.7 | 254.0 | - | 20.8 |
| Montenegro | 64.7 | 352.3 | 23.5 | 141.5 | 131.6 | 41.2 | 79.1 |
| Mauritius | 68.1 | 350.4 | 44.8 | 73.4 | 137.7 | 23.3 | 139.3 |
| Bolivia | 89.8 | 315.6 | 70.4 | 95.5 | 152.2 | 19.4 | 67.8 |
| Afghanistan | 26.7 | 287.5 | 13.9 | 68.3 | 181.7 | 12.8 | 37.5 |
| Cambodia | 52.9 | 278.9 | 38.9 | 89.1 | 140.5 | 14.0 | 49.3 |
| Mozambique | 48.8 | 271.2 | 41.9 | 112.4 | 128.2 | 6.9 | 30.6 |
| Honduras | 14.8 | 254.7 | 10.4 | 36.7 | 160.1 | 4.4 | 57.8 |
| Fiji | 107.6 | 251.3 | 64.3 | 90.4 | 123.5 | 43.3 | 37.4 |
| Togo | 30.4 | 245.4 | 14.3 | 85.6 | 148.1 | 16.2 | 11.7 |
| Myanmar | 34.3 | 238.0 | 18.8 | 61.1 | 59.4 | 15.5 | 117.5 |
| Reunion | 82.3 | 226.5 | 56.9 | 123.7 | 80.5 | 25.4 | 22.3 |
| North Korea | 4.5 | 222.2 | 3.6 | 139.4 | 71.1 | 0.8 | 11.7 |
| Madagascar | 60.2 | 180.9 | 46.7 | 74.8 | 89.0 | 13.5 | 17.1 |
| Guatemala | 44.9 | 172.7 | 30.3 | 60.0 | 80.9 | 14.7 | 31.7 |
| Tajikistan | 33.5 | 164.8 | 28.9 | 51.1 | 93.0 | 4.7 | 20.7 |
| Dominican Republic | 23.9 | 164.4 | 17.0 | 58.5 | 86.3 | 7.0 | 19.6 |
| Mali | 65.9 | 156.5 | 39.1 | 59.6 | 71.7 | 26.8 | 25.1 |
| Bhutan | 18.4 | 153.7 | 13.2 | 44.7 | 77.3 | 5.2 | 31.6 |
| Gabon | 53.5 | 140.7 | 36.3 | 58.2 | 72.1 | 17.2 | 10.4 |
| Sierra Leone | 11.6 | 138.3 | 9.5 | 52.9 | 58.4 | 2.2 | 27.0 |
| Niger | 30.7 | 130.4 | 18.2 | 35.6 | 76.8 | 12.5 | 18.0 |
| Swaziland | 39.3 | 124.1 | 27.5 | 45.2 | 53.4 | 11.8 | 25.5 |
| Papua New Guinea | 60.0 | 123.2 | 34.8 | 48.3 | 47.9 | 25.2 | 27.1 |
| Republic of the Congo | 48.0 | 121.5 | 29.1 | 45.9 | 55.1 | 18.9 | 20.5 |
| El Salvador | 25.7 | 112.6 | 18.8 | 23.5 | 42.5 | 6.9 | 46.5 |
| Guyana | 11.4 | 94.6 | 5.9 | 21.8 | 40.5 | 5.5 | 32.3 |
| Angola | 14.9 | 94.1 | 6.3 | 32.0 | 48.5 | 8.5 | 13.6 |
| Laos | 39.7 | 93.0 | 32.1 | 35.3 | 47.0 | 7.6 | 10.8 |
| Lesotho | 16.5 | 89.0 | 13.9 | 22.6 | 35.0 | 2.6 | 31.4 |
| Gambia | 49.2 | 87.9 | 46.1 | 33.8 | 42.7 | 3.1 | 11.4 |
| Liberia | 3.5 | 86.7 | 2.0 | 31.8 | 37.9 | 1.5 | 17.1 |
| New Caledonia | 64.3 | 86.1 | 61.2 | 51.3 | 21.4 | 3.1 | 13.4 |
| French Guiana | 27.0 | 85.1 | 19.8 | 38.9 | 38.9 | 7.1 | 7.3 |
| Trinidad and Tobago | 29.6 | 81.0 | 14.5 | 19.6 | 25.0 | 15.2 | 36.4 |
| Mauritania | 9.1 | 80.6 | 7.5 | 23.6 | 44.8 | 1.6 | 12.2 |
| Greenland | 20.1 | 75.4 | 18.1 | 40.8 | 18.1 | 2.1 | 16.5 |
| Liechtenstein | 40.6 | 73.2 | 25.5 | 31.4 | 24.1 | 15.1 | 17.7 |
| Grenada | 17.2 | 72.9 | 16.8 | 34.4 | 32.6 | 0.3 | 5.9 |
| Guinea | 20.4 | 72.0 | 13.6 | 26.2 | 36.7 | 6.8 | 9.1 |
| Turkmenistan | 5.1 | 71.8 | 2.8 | 12.6 | 44.1 | 2.3 | 15.1 |
| Chad | 10.6 | 66.7 | 10.0 | 21.1 | 36.1 | 0.6 | 9.5 |
| Monaco | 24.3 | 66.3 | 18.6 | 36.4 | 20.9 | 5.7 | 9.0 |
| Faroe Islands | 5.7 | 65.7 | 4.7 | 38.2 | 19.9 | 1.1 | 7.6 |
| Maldives | 5.0 | 63.8 | 3.0 | 30.3 | 24.1 | 2.0 | 9.3 |
| Nicaragua | 48.3 | 58.9 | 34.7 | 23.0 | 33.5 | 13.6 | 2.4 |
| Burundi | 7.6 | 55.0 | 5.5 | 17.1 | 28.9 | 2.1 | 8.9 |
| Eritrea | 24.9 | 53.2 | 21.6 | 17.9 | 24.7 | 3.3 | 10.6 |
| Haiti | 16.7 | 50.6 | 13.7 | 26.6 | 18.3 | 3.0 | 5.7 |
| Vatican | 10.3 | 50.2 | 6.8 | 11.8 | 27.8 | 3.5 | 10.6 |
| Guam | 34.3 | 47.4 | 17.9 | 24.9 | 19.8 | 16.4 | 2.7 |
| French Polynesia | 17.4 | 45.3 | 14.7 | 28.1 | 13.4 | 2.7 | 3.9 |
| Saint Kitts and Nevis | 5.7 | 44.0 | 4.7 | 19.1 | 22.3 | 1.0 | 2.6 |
| South Sudan | 1.0 | 43.7 | 1.0 | 10.8 | 28.5 | - | 4.5 |
| East Timor | 4.0 | 39.7 | 2.6 | 13.4 | 23.6 | 1.4 | 2.7 |
| Cape Verde | 5.0 | 37.1 | 3.6 | 13.6 | 20.4 | 1.3 | 3.1 |
| Barbados | 28.4 | 35.8 | 14.8 | 12.8 | 14.8 | 13.6 | 8.2 |
| Central African Republic | 14.5 | 34.2 | 8.0 | 15.0 | 13.9 | 6.5 | 5.3 |
| Bahamas | 5.7 | 31.5 | 4.8 | 14.8 | 9.2 | 0.9 | 7.6 |
| Martinique | 24.3 | 30.1 | 14.6 | 17.3 | 11.3 | 9.7 | 1.5 |
| San Marino | 9.9 | 28.1 | 1.9 | 10.8 | 12.3 | 8.0 | 5.0 |
| Djibouti | 4.0 | 27.4 | 2.4 | 8.5 | 13.7 | 1.7 | 5.2 |
| Seychelles | 10.0 | 26.6 | 8.5 | 13.8 | 9.9 | 1.5 | 2.9 |
| Guadeloupe | 22.8 | 24.7 | 14.3 | 11.2 | 11.4 | 8.5 | 2.1 |
| U.S. Virgin Islands | 14.6 | 24.3 | 9.7 | 15.2 | 6.7 | 4.9 | 2.4 |
| Guinea-Bissau | 8.5 | 23.8 | 8.5 | 12.2 | 11.6 | - | - |
| Samoa | 9.6 | 23.7 | 4.6 | 10.3 | 8.0 | 5.0 | 5.4 |
| Suriname | 5.2 | 22.8 | 4.9 | 7.9 | 8.4 | 0.3 | 6.4 |
| Belize | 4.8 | 22.7 | 4.8 | 10.7 | 4.6 | - | 7.4 |
| Vanuatu | 5.8 | 19.8 | 4.3 | 7.7 | 7.3 | 1.5 | 4.7 |
| Curacao | - | 19.0 | 6.2 | 11.0 | 1.8 |  |  |
| Solomon Islands | 7.6 | 18.7 | 5.1 | 9.5 | 7.7 | 2.5 | 1.6 |
| Antigua and Barbuda | 3.0 | 16.5 | 0.1 | 5.6 | 6.7 | 2.9 | 4.2 |
| Gibraltar | 3.4 | 16.4 | 1.3 | 7.4 | 8.1 | 2.1 | 0.9 |
| Sao Tome and Principe | 1.0 | 14.2 | 1.0 | 7.4 | 5.9 | - | 0.9 |
| Cayman Islands | 10.1 | 13.8 | 5.8 | 5.9 | 7.6 | 4.3 | 0.2 |
| Bermuda | 17.0 | 12.6 | 15.1 | 7.2 | 4.8 | 1.8 | 0.7 |
| Andorra | 7.2 | 12.1 | 5.2 | 2.9 | 7.0 | 2.0 | 2.2 |
| Comoros | 1.4 | 11.6 | 0.2 | 6.2 | 3.4 | 1.2 | 2.0 |
| Aruba | - | 10.6 | 6.6 | 4.0 | - |  |  |
| Micronesia | 3.1 | 10.5 | 3.1 | 1.5 | 2.6 | - | 6.3 |
| Jersey | 8.7 | 9.4 | 7.8 | 3.9 | 2.4 | 0.9 | 3.1 |
| Anguilla | 0.2 | 7.9 | 0.2 | 2.1 | 5.7 | - | - |
| Palau | 6.8 | 7.2 | 2.8 | 5.1 | 1.5 | 4.0 | 0.6 |
| Dominica | 2.1 | 7.1 | 1.1 | 2.4 | 4.1 | 1.0 | 0.7 |
| American Samoa | 4.0 | 6.3 | 4.0 | 1.8 | 2.8 | - | 1.8 |
| Falkland Islands | 11.2 | 6.3 | 10.2 | 3.1 | 2.1 | 1.0 | 1.0 |
| Equatorial Guinea | 4.0 | 6.2 | 3.2 | 3.5 | 1.6 | 0.8 | 1.1 |
| Saint Vincent and the Grenadines | 0.7 | 5.5 | 0.7 | 2.6 | 0.2 | - | 2.7 |
| Tonga | 4.7 | 5.1 | 1.7 | 3.3 | 1.8 | 3.0 | - |
| United States Minor Outlying Islands | - | 5.0 | - | - | 5.0 |  |  |
| Netherlands Antilles | 12.3 | 4.3 | 11.6 | 2.8 | 1.6 | 0.8 | - |
| Montserrat | 3.2 | 4.3 | 2.3 | 3.9 | 0.4 | 1.0 | - |
| Saint Lucia | 4.0 | 4.0 | 2.6 | 2.8 | 1.0 | 1.3 | 0.2 |
| Cook Islands | 0.7 | 3.2 | 0.7 | 0.4 | 2.7 | - | 0.1 |
| British Virgin Islands | 3.1 | 3.2 | 2.1 | 0.1 | 1.3 | 1.0 | 1.8 |
| Mayotte | 3.8 | 3.2 | 1.5 | 1.1 | 2.1 | 2.3 | 0.1 |
| Kiribati | 0.9 | 2.9 | 0.4 | 0.5 | 1.1 | 0.5 | 1.2 |
| Norfolk Island | - | 2.6 | 0.3 | 0.3 | 2.0 |  |  |
| Saint Helena | 0.1 | 2.6 | 0.1 | 1.6 | 0.1 | - | 0.9 |
| British Indian Ocean Territory | - | 2.4 | 2.1 | 0.1 | 0.2 |  |  |
| Niue | 2.0 | 1.7 | - | 1.2 | 0.5 | 2.0 | - |
| South Georgia and the South Sandwich Islands | - | 1.7 | - | 0.6 | 1.1 |  |  |
| Tuvalu | 0.8 | 1.6 | 0.8 | 0.6 | 1.0 | - | - |
| Turks and Caicos Islands | 1.8 | 1.5 | 1.3 | 0.6 | 0.9 | 0.5 | - |
| Northern Mariana Islands | 0.8 | 1.5 | 0.8 | 1.0 | 0.5 | - | - |
| Marshall Islands | 4.1 | 1.3 | 3.6 | 0.2 | 1.1 | 0.5 | - |
| Serbia and Montenegro | 17.4 | 1.2 | 16.4 | 1.0 | 0.2 | 1.0 | - |
| Bouvet Island | - | 1.0 | - | 1.0 | - |  |  |
| Sint Maarten | - | 0.9 | 0.3 | 0.6 | - |  |  |
| Svalbard and Jan Mayen | - | 0.9 | 0.9 | - | - |  |  |
| Antarctica | 1.5 | 0.9 | 0.5 | 0.0 | 0.5 | 1.0 | 0.3 |
| Pitcairn | - | 0.7 | - | 0.7 | - |  |  |
| Saint Martin | - | 0.5 | 0.1 | 0.0 | 0.4 |  |  |
| Tokelau | - | 0.5 | 0.5 | - | - |  |  |
| Saint Pierre and Miquelon | - | 0.5 | - | 0.5 | - |  |  |
| Nauru | 0.3 | 0.0 | 0.3 | 0.0 | - | - | - |
| Cocos Islands | 0.8 | - | - |  | 0.8 |  |  |
| Christmas Island | 0.5 | - | 0.5 |  | - |  |  |

**Supplementary Figure 1.** Top-producing countries with fractional count of publications in 2006 and 2024 broken down according to type of venue


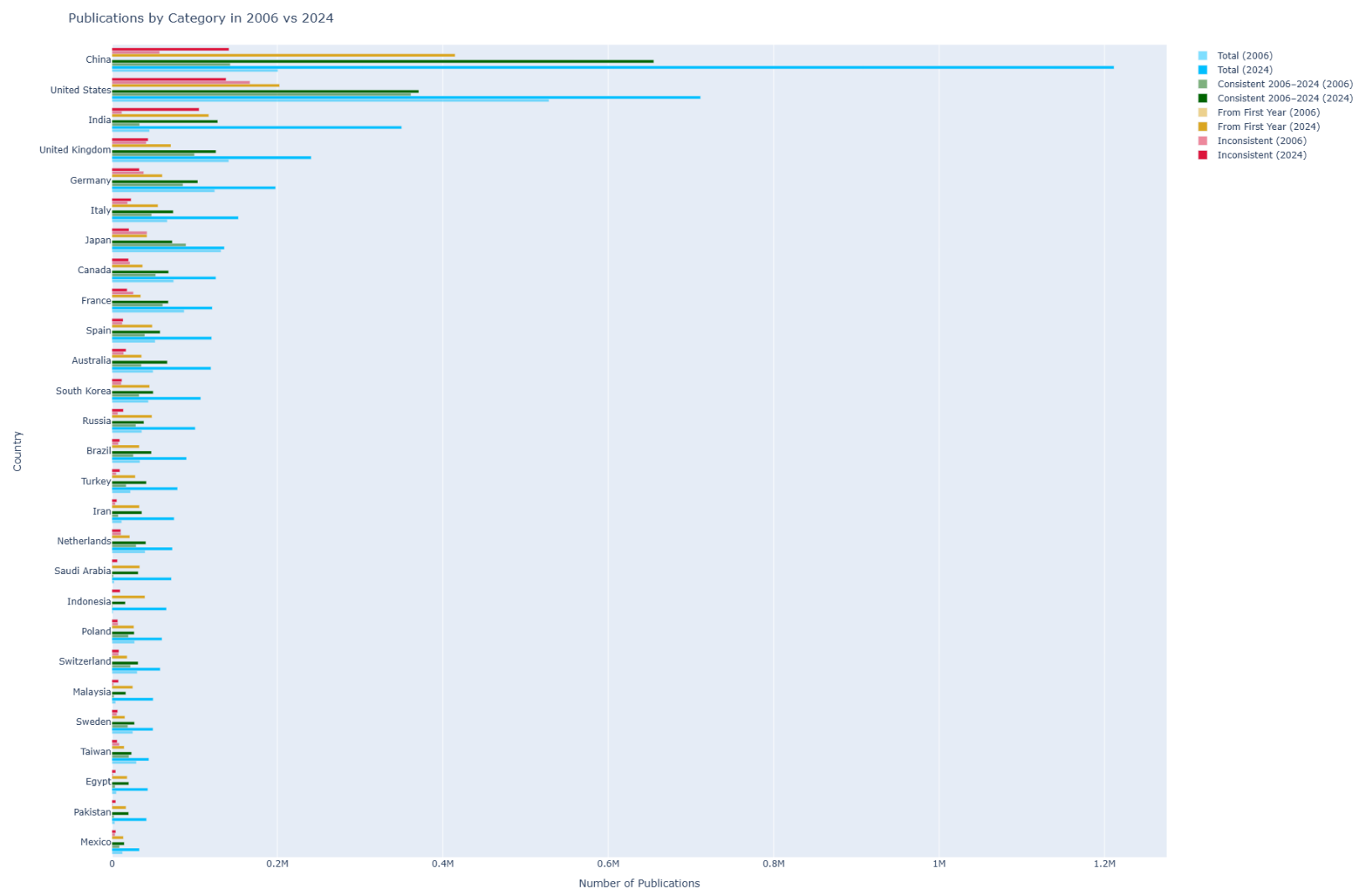

Supplement: Supplementary file 1 — Supplementary Material 1. [file 41073_2026_190_MOESM1_ESM.docx]
